# Supplementary figures and images for: Genetic context modulates aging and degeneration in the murine retina
Source: Mol Neurodegener. 2025 Jan 20;20:8. doi: 10.1186/s13024-025-00800-9 (PMC11744848; doi:10.1186/s13024-025-00800-9)

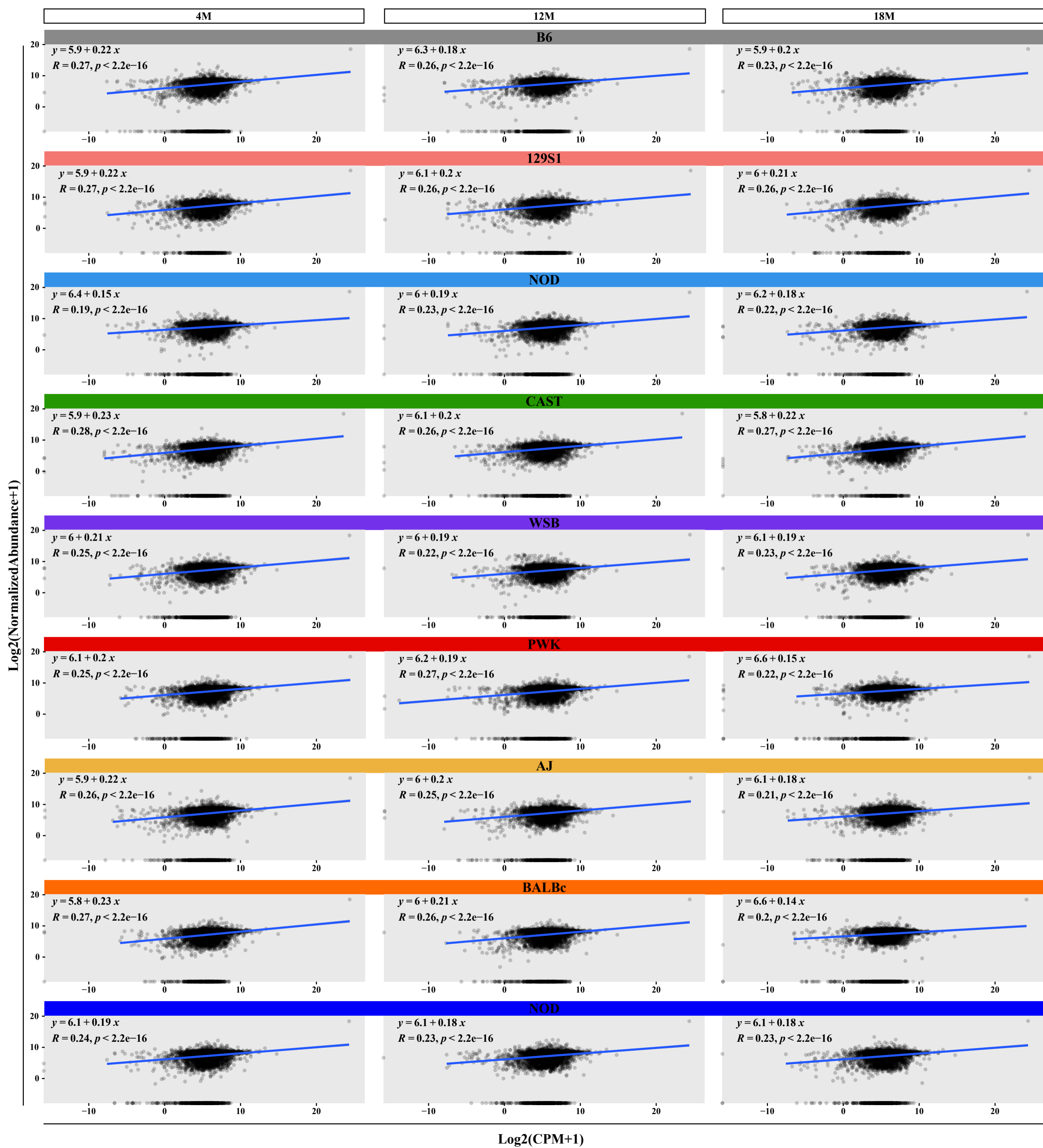

Supplement: Supplementary file 1 — Supplementary Material 1. [file 13024_2025_800_MOESM1_ESM.pdf]

epiretinal membrane

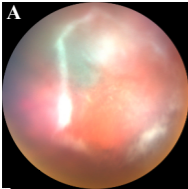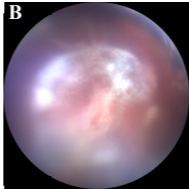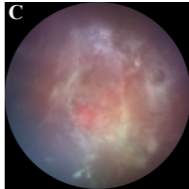

Supplement: Supplementary file 3 — Supplementary Material 3. [file 13024_2025_800_MOESM3_ESM.pdf]

# WSB 18M

20X

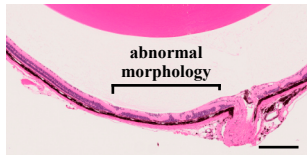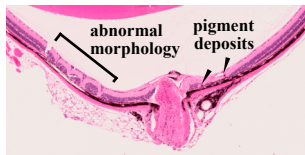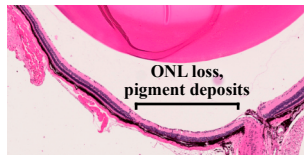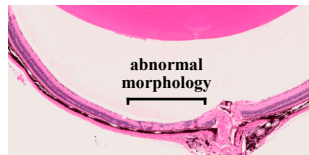

40X

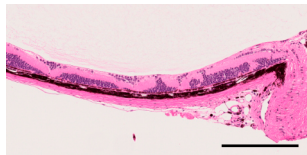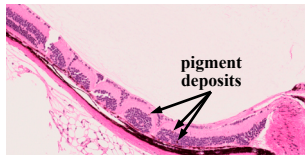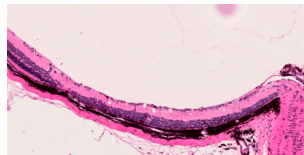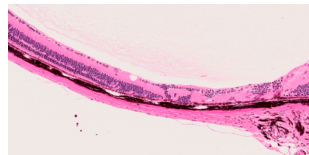

80X

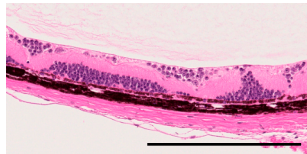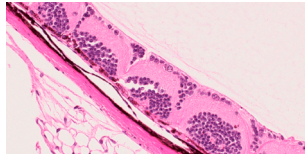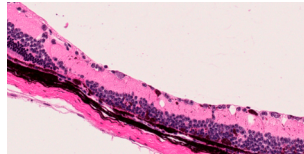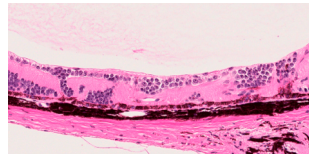

Supplement: Supplementary file 4 — Supplementary Material 4. [file 13024_2025_800_MOESM4_ESM.pdf]

# WSB 18M

20X

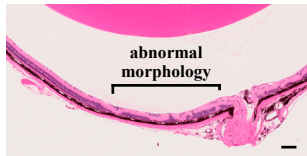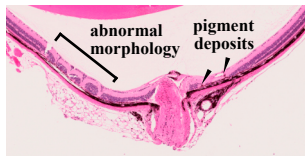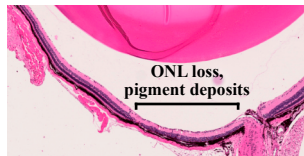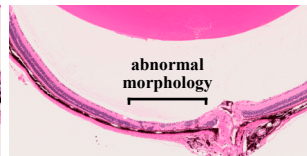

40X

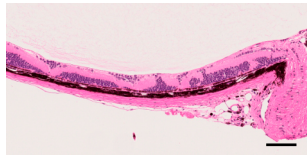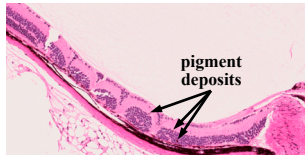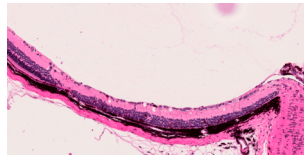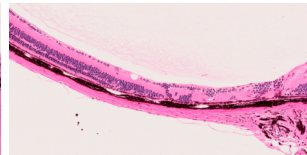

80X

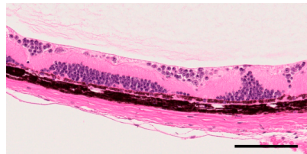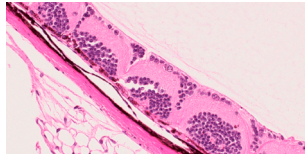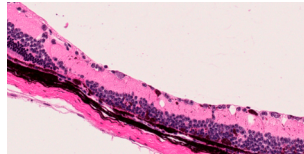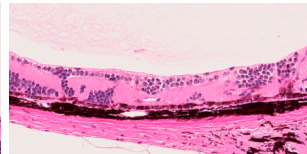

Supplement: Supplementary file 5 — Supplementary Material 5. [file 13024_2025_800_MOESM5_ESM.pdf]

Fluorescein angiography

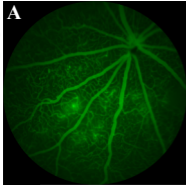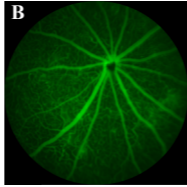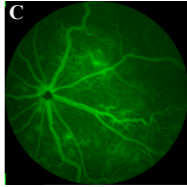

Fundus

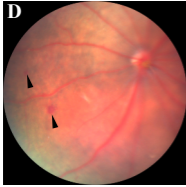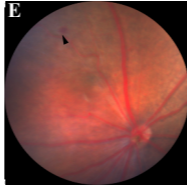

Prussian Blue histology

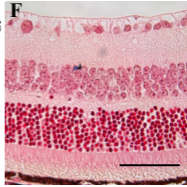

Supplement: Supplementary file 7 — Supplementary Material 7. [file 13024_2025_800_MOESM7_ESM.pdf]
