# Supplementary material for: Genetic context modulates aging and degeneration in the murine retina
Source: Mol Neurodegener. 2025 Jan 20;20:8. doi: 10.1186/s13024-025-00800-9 (PMC11744848; doi:10.1186/s13024-025-00800-9)
Supplement: Supplementary file 2 — Supplementary Material 2. [file 13024_2025_800_MOESM2_ESM.pdf]

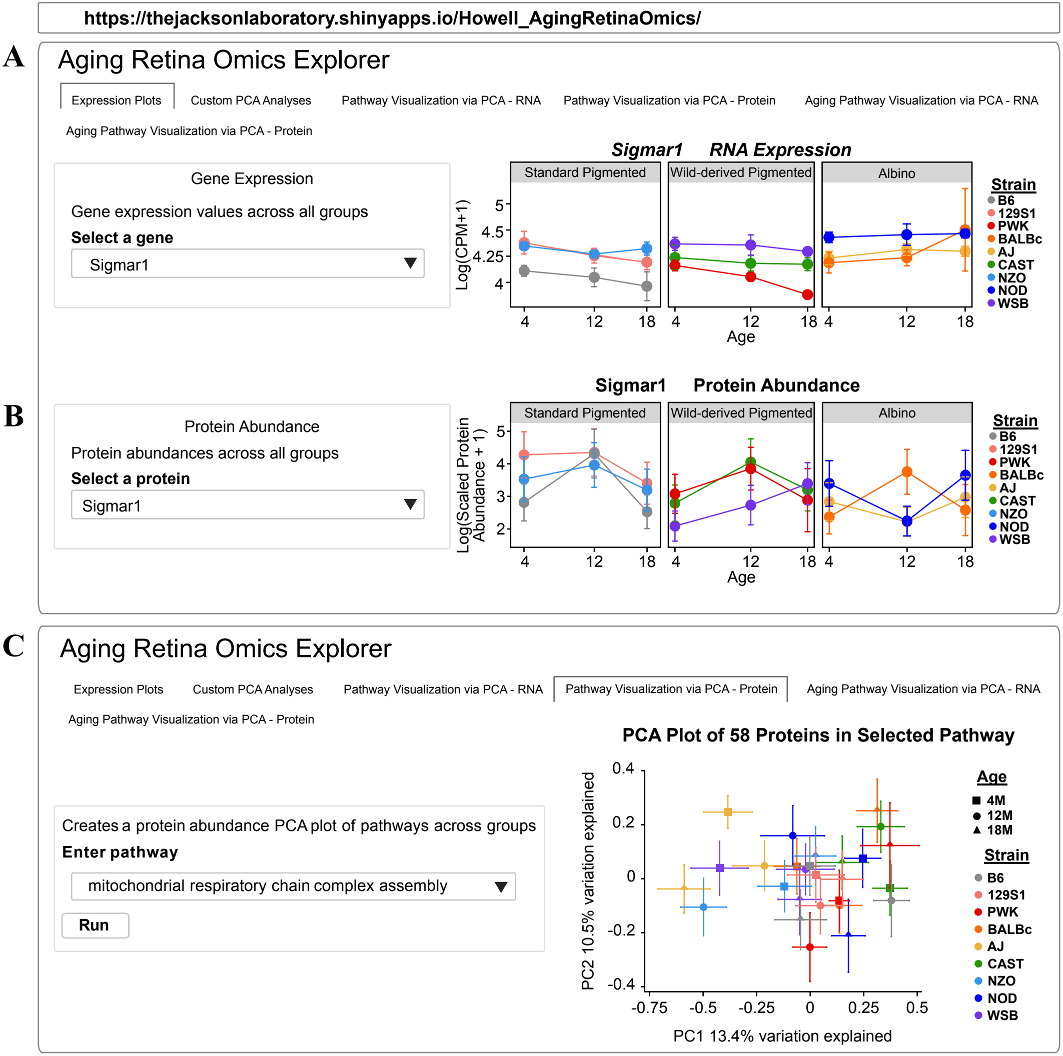
https://thejacksonlaboratory.shinyapps.io/Howell_AgingRetinaOmics/
A
Aging Retina Omics Explorer
Expression Plots
Custom PCA Analyses
Pathway Visualization via PCA - RNA
Pathway Visualization via PCA - Protein
Aging Pathway Visualization via PCA - RNA
Aging Pathway Visualization via PCA - Protein
Gene Expression
Gene expression values across all groups
Select a gene
Sigmar1
Sigmar1 RNA Expression
Standard Pigmented
Wild-derived Pigmented
Albino
Log(CPM+1)
Age
Strain
B6
129S1
PWK
BALBc
AJ
CAST
NZO
NOD
WSB
B
Protein Abundance
Protein abundances across all groups
Select a protein
Sigmar1
Sigmar1 Protein Abundance
Log(Scaled Protein Abundance + 1)
C
Aging Retina Omics Explorer
Creates a protein abundance PCA plot of pathways across groups
Enter pathway
mitochondrial respiratory chain complex assembly
Run
PCA Plot of 58 Proteins in Selected Pathway
PC2 10.5% variation explained
PC1 13.4% variation explained
Age
4M
12M
18M
