# Supplementary material for: Genetic context modulates aging and degeneration in the murine retina
Source: Mol Neurodegener. 2025 Jan 20;20:8. doi: 10.1186/s13024-025-00800-9 (PMC11744848; doi:10.1186/s13024-025-00800-9)
Supplement: Supplementary file 6 — Supplementary Material 6. [file 13024_2025_800_MOESM6_ESM.pdf]

A. Protein-protein interaction network 4M WSB v Pigmented Strains

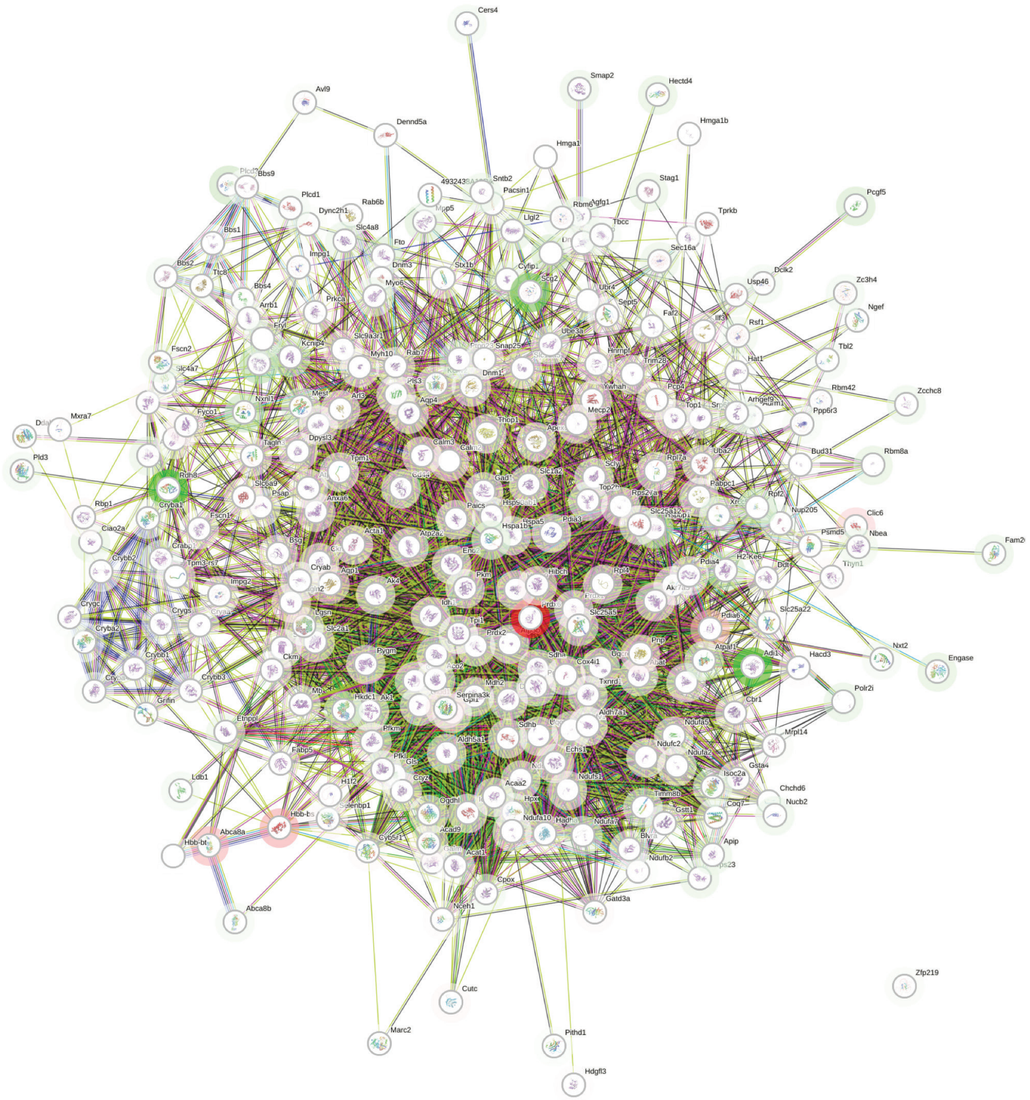

B. Top 25 Enriched GO terms

GO Term Category	GO Term	FDR
Component	Myelin sheath	1.20E-06
Component	Mitochondrion	0.00029
Process	Generation of precursor metabolites and energy	0.00038
Compartments	Oxidoreductase complex	0.00059
Compartments	Mitochondrion	0.00085
Compartments	Respiratory chain complex	0.00099
Process	Organic acid metabolic process	0.0032
Process	Aerobic respiration	0.0032
Process	Purine ribonucleotide metabolic process	0.0032
Process	Purine ribonucleoside triphosphate metabolic process	0.0032
Process	Energy derivation by oxidation of organic compounds	0.0032
Process	Oxoacid metabolic process	0.0032
Process	Small molecule metabolic process	0.0032
Process	Cellular respiration	0.0032
Process	ATP metabolic process	0.0032
Process	Carboxylic acid metabolic process	0.0037
Process	Small molecule catabolic process	0.0037
Process	Sensory perception	0.0044
Process	Lens development in camera-type eye	0.0079
Process	Response to xenobiotic stimulus	0.0079
Process	Dicarboxylic acid metabolic process	0.0079
Process	Homeostatic process	0.0091
Compartments	Mitochondrial inner membrane	0.0094
Component	Transmembrane transporter complex	0.0098
Component	Oxidoreductase complex	0.01
