# Supplementary material for: Genetic context modulates aging and degeneration in the murine retina
Source: Mol Neurodegener. 2025 Jan 20;20:8. doi: 10.1186/s13024-025-00800-9 (PMC11744848; doi:10.1186/s13024-025-00800-9)
Supplement: Supplementary file 8 — Supplementary Material 8. [file 13024_2025_800_MOESM8_ESM.pdf]

A. Protein-protein interaction network 4M NZO v Pigmented Strains

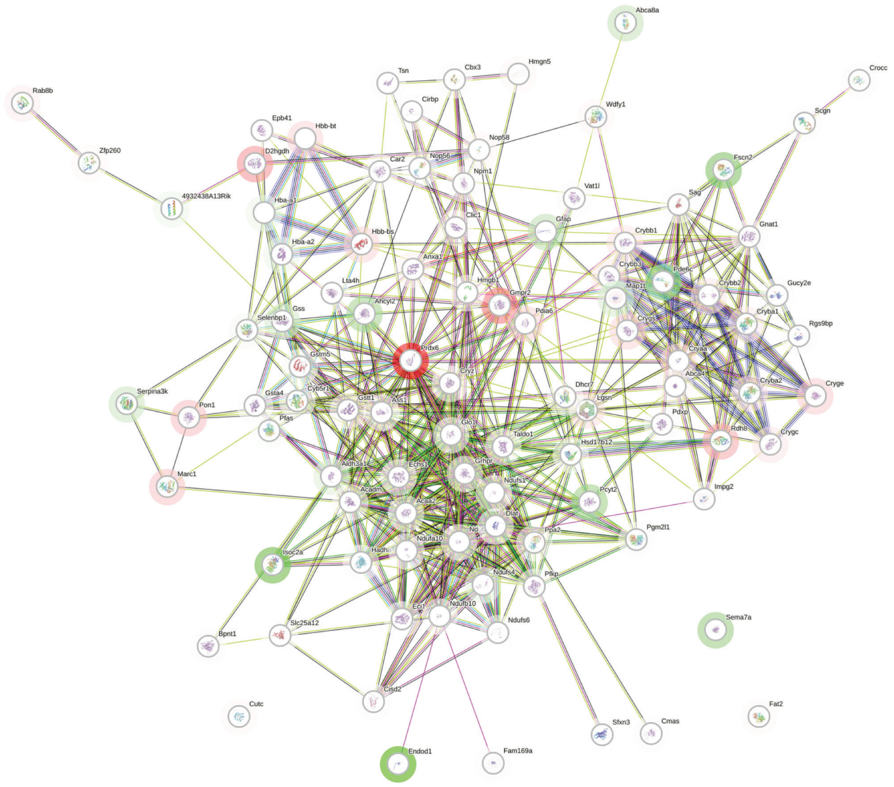

B. Enriched GO terms

GO Term Category	GO Term	FDR
Process	Visual perception	1.81E-07
Process	Lens development in camera-type eye	7.77E-05
Process	Small molecule metabolic process	8.05E-05
Process	Glutathione metabolic process	0.00077
Process	Cellular modified amino acid metabolic process	0.0028
Process	Eye development	0.0028
Process	Response to toxic substance	0.0028
Process	Gas transport	0.0111
Process	Sulfur compound metabolic process	0.0111
Process	Camera-type eye development	0.0137
Process	Cellular detoxification	0.0179
Process	Oxoacid metabolic process	0.0179
Component	Hemoglobin complex	0.0242
Process	Organophosphate metabolic process	0.039
Compartment	Hemoglobin complex	0.0461
Compartment	Oxidoreductase complex	0.0461
Process	Cellular aldehyde metabolic process	0.0486
