# Supplementary material for: Genetic context modulates aging and degeneration in the murine retina
Source: Mol Neurodegener. 2025 Jan 20;20:8. doi: 10.1186/s13024-025-00800-9 (PMC11744848; doi:10.1186/s13024-025-00800-9)
Supplement: Supplementary file 13 — Supplementary Material 13. [file 13024_2025_800_MOESM13_ESM.docx]

**Supplementary Materials**

*Genetic context modulates aging and degeneration in the murine retina*

Olivia J. Marola^1*^, Michael MacLean^1*^, Travis L. Cossette^1^, Cory A. Diemler^1,3^, Amanda A. Hewes^1^, Alaina M. Reagan^1^, Jonathan Nyandu Kanyinda^1^, Daniel A. Skelly^1^, and Gareth R. Howell^1,2,3#^

^1^The Jackson Laboratory, Bar Harbor, ME 04609, USA

^2^Sackler School of Graduate Biomedical Sciences, Tufts University School of Medicine, Boston, MA 02111, USA

^3^Graduate School of Biomedical Sciences and Engineering, University of Maine, Orono, ME 04469, USA

^*^These authors contributed equally.

Correspondence:^[#](mailto:#gareth.howell@jax.org)^[gareth.howell@jax.org](mailto:#gareth.howell@jax.org)

**This supplementary file includes:**

*Additional File 1. Supplemental Figure 1. Correlation of corresponding proteomics and transcriptomics data across strains and ages.*

*Additional File 2. Supplemental Figure 2. Development of a user driven query-based web-tool.*

*Additional File 3. Supplemental Figure 3. Aged NZO mice develop epiretinal membranes.*

*Additional File 4. Supplemental Figure 4. WSB mice exhibit abnormal retinal structures by histology.*

*Additional File 5. Supplemental Figure 5:* *Immunohistochemistry of rhodopsin and red/green cone opsin.*

*Additional File 6. Supplemental Figure 6:* *4M WSB mice altered photoreceptor outer segment and metabolic DEPs relative to pigmented mice.*

*Additional File 7. Supplemental Figure 7. NZO mice develop microaneurysms and hemorrhages.*

*Additional File 8. Supplemental Figure 8:* *4M NZO mice exhibit reduced expression of antioxidant pathway proteins relative to pigmented strains.*

*Additional File 9. Supplemental Table 1.* Excel file containing diagram of total numbers of mice used for transcriptomics and proteomics by strain, age, and sex. Additional tabs include individual sample metadata for proteomics and transcriptomics.

*Additional File 10. Supplemental Table 2.* Excel sheets containing the common aging signature DEG and DEP analysis results, and the results of GO enrichment analyses for both the common aging signature DEGs and DEPs. For **DEG** analyses: *N*=8 (4F,4M) NZO, 129S1, and B6 mice at each age*;* NOD mice: *N*=8 (4F,4M) at 4M*, N*=7 (3F,4M) at 12M, *N*=6 (2F,4M) at 18M*;* AJ mice: *N*=8 (4F,4M) at 4M and 12M, *N*=7 (4F,3M) at 18M; BALBc mice: *N*=7 (3F,4M) at 4M and 12M, *N*=4F at 18 M; PWK mice: *N*=8 (4F,4M) at 4M and 12M, *N*=3F at 18M; CAST mice: *N*=8 (4F,4M) at 4M and 12M, *N=7* (3F,4M) at 18M; WSB mice: *N=7* (3F,4M) at 4M, *N*=8 (4F,4M) at 12M and 18M. For **DEP** analyses: *N*=8 (4F,4M) NZO, 129S1, and CAST mice at each age*;* NOD mice: *N*=7 (4F,3M) at 4M*, N*=8 (4F,4M) at 12M, *N*=6 (2F,4M) at 18M*;* AJ mice: *N*=8 (4F,4M) at 4M and 12M, *N*=7 (4F,3M) at 18M; BALBc mice: *N*=7 (3F,4M) at 4M, *N*=8 (4F,4M) at 12M, *N*=4F at 18 M; PWK mice: *N*=8 (4F,4M) at 4M and 12M, *N*=3F at 18M; B6 mice: *N*=8 (4F,4M) at 4M and 18M, *N=7* (3F,4M) at 12M; WSB mice: *N=7* (3F,4M) at 4M, *N*=7 (4F,3M) at 12M, *N*=8 (4F,4M) at 18M.

*Additional File 11. Supplemental Table 3.* Excel sheets containing the proteomics analysis of DEPs for 4M WSB vs the other pigmented strains, and the results of STRING-dB protein-protein interaction analysis and GO term enrichment. *N*=8 (4F,4M) NZO, 129S1, PWK, B6 and CAST mice*;* WSB mice: *N=7* (3F,4M).

*Additional File 12. Supplemental Table 4.* Excel sheets containing the proteomics analysis of DEPs for 4M NZO vs the other pigmented strains, and the results of STRING-dB protein-protein interaction analysis and GO term enrichment. *N*=8 (4F,4M) NZO, 129S1, PWK, B6 and CAST mice*;* WSB mice: *N=7* (3F,4M).

**

*Supplemental Figure 1. Correlation of corresponding proteomics and transcriptomics data across strains and ages.*

Scatter plots showing Log2(Normalized Abundance+1) of proteins on the y-axis and Log2(Counts per million mapped reads + 1) of corresponding transcripts on the x-axis. A line of best fit was calculated for each strain at each age and reported with R and p-values. For the transcriptional data (x-axis): *N*=8 (4F,4M) NZO, 129S1, and B6 mice at each age*;* NOD mice: *N*=8 (4F,4M) at 4M*, N*=7 (3F,4M) at 12M, *N*=6 (2F,4M) at 18M*;* AJ mice: *N*=8 (4F,4M) at 4M and 12M, *N*=7 (4F,3M) at 18M; BALBc mice: *N*=7 (3F,4M) at 4M and 12M, *N*=4F at 18 M; PWK mice: *N*=8 (4F,4M) at 4M and 12M, *N*=3F at 18M; CAST mice: *N*=8 (4F,4M) at 4M and 12M, *N=7* (3F,4M) at 18M; WSB mice: *N=7* (3F,4M) at 4M, *N*=8 (4F,4M) at 12M and 18M. For the proteomics data (y-axis): *N*=8 (4F,4M) NZO, 129S1, and CAST mice at each age*;* NOD mice: *N*=7 (4F,3M) at 4M*, N*=8 (4F,4M) at 12M, *N*=6 (2F,4M) at 18M*;* AJ mice: *N*=8 (4F,4M) at 4M and 12M, *N*=7 (4F,3M) at 18M; BALBc mice: *N*=7 (3F,4M) at 4M, *N*=8 (4F,4M) at 12M, *N*=4F at 18 M; PWK mice: *N*=8 (4F,4M) at 4M and 12M, *N*=3F at 18M; B6 mice: *N*=8 (4F,4M) at 4M and 18M, *N=7* (3F,4M) at 12M; WSB mice: *N=7* (3F,4M) at 4M, *N*=7 (4F,3M) at 12M, *N*=8 (4F,4M) at 18M.

**

*Supplemental Figure 2. Development of a user driven query-based web-tool.*

Screen captures of publicly-accessible webtool (https://thejacksonlaboratory.shinyapps.io/Howell_AgingRetinaOmics/). Illustration of searching and plotting for the gene “*Sigmar1”* for RNA levels (**A**) or corresponding protein levels (**B**) across strains and ages. **C.** Illustration of plotting a principal component plot using the proteins detected in the proteomics dataset for the “mitochondrial respiratory chain complex assembly” GO term. For the transcriptional data (x-axis): *N*=8 (4F,4M) NZO, 129S1, and B6 mice at each age*;* NOD mice: *N*=8 (4F,4M) at 4M*, N*=7 (3F,4M) at 12M, *N*=6 (2F,4M) at 18M*;* AJ mice: *N*=8 (4F,4M) at 4M and 12M, *N*=7 (4F,3M) at 18M; BALBc mice: *N*=7 (3F,4M) at 4M and 12M, *N*=4F at 18 M; PWK mice: *N*=8 (4F,4M) at 4M and 12M, *N*=3F at 18M; CAST mice: *N*=8 (4F,4M) at 4M and 12M, *N=7* (3F,4M) at 18M; WSB mice: *N=7* (3F,4M) at 4M, *N*=8 (4F,4M) at 12M and 18M. For the proteomics data (y-axis): *N*=8 (4F,4M) NZO, 129S1, and CAST mice at each age*;* NOD mice: *N*=7 (4F,3M) at 4M*, N*=8 (4F,4M) at 12M, *N*=6 (2F,4M) at 18M*;* AJ mice: *N*=8 (4F,4M) at 4M and 12M, *N*=7 (4F,3M) at 18M; BALBc mice: *N*=7 (3F,4M) at 4M, *N*=8 (4F,4M) at 12M, *N*=4F at 18 M; PWK mice: *N*=8 (4F,4M) at 4M and 12M, *N*=3F at 18M; B6 mice: *N*=8 (4F,4M) at 4M and 18M, *N=7* (3F,4M) at 12M; WSB mice: *N=7* (3F,4M) at 4M, *N*=7 (4F,3M) at 12M, *N*=8 (4F,4M) at 18M. All error bars represent SEM.

**

*Supplemental Figure 3. Aged NZO mice develop epiretinal membranes.*

**A-C.** Fundus images of epiretinal membranes we detected exclusively in 18M NZO mice.

*Supplemental Figure 4. WSB mice exhibit abnormal retinal structures by histology.*

Four representative examples of abnormal retinal structures identified in aged WSB animals, which were never observed in young mice. Scale bars, 100µm.

*Supplemental Figure 5. Immunohistochemistry of rhodopsin and red/green cone opsin.*

Representative images of (**A**) rhodopsin (RHO) and (**B**) red/green cone opsin (OPN1LW) staining. Inner and outer segment (IS/OS) is noted with a bracket. **C**. Quantification of the width of the IS/OS by retinal location normalized to the relative change in retinal length compared to 4M controls. **D**. Quantification of the positive RHO area normalized to the relative change in retinal length compared to 4M controls. **E**. Quantification of the number of OPN1LW+ cells per normalized to % change in retina length from respective mean 4M baseline in Main Figure 8D . In **C-E**: mixed effects or two-way ANOVA analyses with repeated measures were used to assess regional and aging effects within strains with corresponding post-hoc Tukey’s multiple comparison tests. Error bars represent SEM. In **A, C, D**: for B6: *N*=7 eyes at 4M, 3-4 at 12M, 5-6 at 18M; for NZO: *N*=7 eyes at 4M, 5-6 at 12M, 7-8 at 18M; for WSB: *N*=8-9 eyes at 4M, 5-8 at 12M, 6-7 at 18M. In **B,E**: for B6: *N*=4 eyes at 4M, 6 at 12M, 7 at 18M; for NZO: *N*=7 eyes at 4M, 6 at 12M, 7 at 18M; for WSB: *N*=8 eyes at 4M, 6 at 12M, 6-8 at 18M. Scale bars, 50µm.

**

*Supplemental Figure 6:* *4M WSB mice altered photoreceptor outer segment and metabolic DEPs relative to pigmented mice.*

**A**. Physical STRING protein-protein interaction network of DEPs associated with 4M WSB vs other pigmented strains. 258 total proteins, 3247 interactions, *p*<0.0001*.* Node glow is colored by log_2_(fold-change) magnitude. Lines indicate physical interactions. **B**. Top 25 Enriched GO terms with FDR < 0.05 associated with the DEPs identified between 4M WSB and the other pigmented strains. *N*=8 (4F,4M) NZO, 129S1, PWK, B6 and CAST mice*;* WSB mice: *N=7* (3F,4M).

**

*Supplemental Figure 7. NZO mice develop microaneurysms and hemorrhages.*

**A-C.** Representative fluorescein angiography images of microaneurysms, and hemorrhages in 12-18M NZO mice. Notable vessel tortuosity is also visible. **D-E.** Fundus images of NZO mice showing deep red spots suggestive of hemorrhages. **F.** Prussian blue histological staining of an NZO retina indicating a hemorrhage event within the inner nuclear layer. Nuclei are stained pink with Fast Red. Scale bar: 50µm.

*Supplemental Figure 8:* *4M NZO mice exhibit reduced expression of antioxidant pathway proteins relative to pigmented strains.*

**A**. Physical STRING protein-protein interaction network of DEPs associated with 4M NZO vs other pigmented strains. 97 total proteins, 488 interactions, *p*<0.0001*.* Node glow is colored by log_2_(fold-change) magnitude. Lines indicate physical interactions. **B**. Enriched GO terms with FDR < 0.05 associated with the DEPs identified between 4M NZO and other pigmented strains. *N*=8 (4F,4M) NZO, 129S1, PWK, B6 and CAST mice*;* WSB mice: *N=7* (3F,4M).
